# Durable control of psoriatic arthritis with guselkumab across domains and patient characteristics: post hoc analysis of a phase 3 study

**DOI:** 10.1007/s10067-024-06991-8

**Published:** 2024-06-07

**Authors:** Christopher T. Ritchlin, Philip J. Mease, Wolf-Henning Boehncke, John Tesser, Soumya D. Chakravarty, Emmanouil Rampakakis, May Shawi, Elena Schiopu, Joseph F. Merola, Iain B. McInnes, Atul Deodhar

**Affiliations:** 1 University of Rochester Medical Center, Rochester, NY USA; 2 Rheumatology Research, Providence Swedish Medical Center, Seattle, WA USA; 3 Division of Dermatology and Venereology, Geneva University Hospitals, University of Geneva, Geneva, Switzerland; 4 Arizona Arthritis & Rheumatology Associates, P.C., Phoenix, AZ USA; 5 Janssen Scientific Affairs, LLC, a Johnson & Johnson Company, Horsham, PA USA; 6 Drexel University College of Medicine, Philadelphia, PA USA; 7 Department of Pediatrics, McGill University, Montreal, QC Canada; 8 Scientific Affairs, JSS Medical Research, Inc, Montreal, QC Canada; 9 Janssen Research & Development, LLC, Titusville, NJ USA; 10 Medical College of Georgia at Augusta University, Augusta, GA USA; 11 Department of Dermatology and Department of Medicine, Division of Rheumatology, UT Southwestern Medical Center, Dallas, TX USA; 12 College of Medical Veterinary and Life Sciences, University of Glasgow, Glasgow, UK; 13 Division of Arthritis and Rheumatic Diseases, Oregon Health & Science University, Portland, OR USA; 14 University of Washington School of Medicine, Seattle, WA USA

**Keywords:** Disease control, Domain, Guselkumab, Patient-reported outcome, Psoriatic arthritis

## Abstract

**Objectives:**

Evaluate patterns of stringent disease control with 2 years of guselkumab across key disease-identified domains and patient-reported outcomes (PROs) in subgroups of patients with psoriatic arthritis (PsA) defined by baseline characteristics.

**Method:**

This post hoc analysis of DISCOVER-2 (Clinicaltrials.gov NCT03158285) evaluated biologic-naïve PsA patients (≥ 5 swollen/ ≥ 5 tender joints, C-reactive protein [CRP] ≥ 0.6 mg/dL) randomized to guselkumab every 4 weeks (Q4W); guselkumab at Weeks 0 and 4, then Q8W; or placebo with crossover to guselkumab Q4W at Week 24. Achievement of American College of Rheumatology 50/70% improvement (ACR50/70), Investigator’s Global Assessment (IGA) 0, dactylitis/enthesitis resolution, Functional Assessment of Chronic Illness Therapy (FACIT)-Fatigue response (≥ 4-point improvement), HAQ-Disability Index (HAQ-DI) response (≥ 0.35-point improvement), PsA Disease Activity Score (PASDAS) low disease activity (LDA), and minimal disease activity (MDA) was assessed at Weeks 24, 52, and 100 in subgroups defined by sex and baseline medication use, body mass index, PsA duration, swollen/tender joints, CRP, and psoriasis severity/extent. Patients with missing categorical response data were considered nonresponders.

**Results:**

442/493 (90%) guselkumab-randomized patients completed treatment through Week 100. Significant multi-domain efficacy of guselkumab versus placebo was shown across adequately sized patient subgroups. A pattern of continuous improvement was observed across key PsA domains and PROs within patient subgroups: 65%–85% of guselkumab-randomized patients had enthesitis/dactylitis resolution, 50%–70% achieved complete skin clearance, 60%–80% reported meaningful improvements in function/fatigue, 40%–65% achieved PASDAS LDA, and 35%–50% achieved MDA at Week 100.

**Conclusion:**

Patients with active PsA receiving guselkumab demonstrated durable achievement of stringent endpoints associated with disease control across key PsA domains and PROs, regardless of baseline characteristics.

**Table:** 

**Key Points** *• Among biologic-naïve patients with highly active psoriatic arthritis (PsA), efficacy of guselkumab across stringent disease endpoints and patient-reported outcomes (PROs) at Week 24 was consistent regardless of baseline demographics and disease characteristics.* *• Within guselkumab-randomized PsA patient subgroups, major improvements in joint disease activity, complete skin clearance, dactylitis/enthesitis resolution, clinically meaningful improvements in PROs, and achievement of low overall disease activity were maintained through Week 100.* *• Durable stringent endpoint achievement indicating disease control was observed with guselkumab, regardless of baseline patient or disease characteristics.*

**Graphical Abstract:**

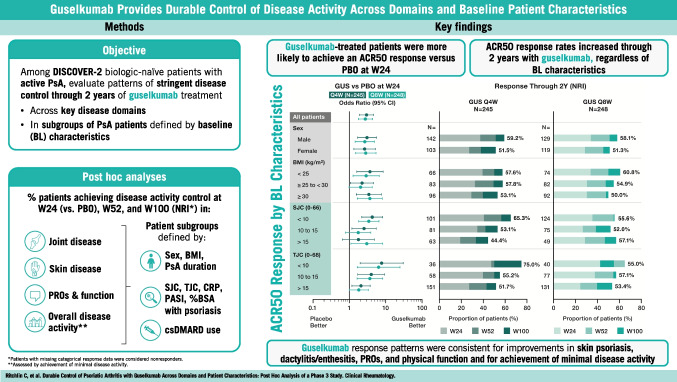

**Supplementary Information:**

The online version contains supplementary material available at 10.1007/s10067-024-06991-8.

## Introduction

PsA is a multi-domain, chronic inflammatory condition [1]. The most recent treatment recommendations from the Group for Research and Assessment of Psoriasis and Psoriatic Arthritis (GRAPPA) emphasize the importance of selecting treatments that target the six key domains of PsA (peripheral arthritis, axial disease, enthesitis, dactylitis, and skin and nail psoriasis) according to disease activity in individual patients [2]. Related conditions and comorbidities, including inflammatory bowel disease (IBD) and uveitis, should also be considered [2]. While biologic therapies generally offer greater improvement in PsA symptoms than conventional synthetic (cs)DMARDs in patients with active PsA [2, 3], the heterogeneity of PsA can lead to varying initial and long-term responses to treatments. Variable responses to TNF, IL-17A, IL-12/23, Janus kinase (JAK), and phosphodiesterase-4 (PDE-4) inhibitors (i) have also been shown in patient subgroups defined by baseline characteristics [4–9]. In several observational studies female sex and obesity were associated with lower efficacy in response to TNFi in patients with PsA [4, 5]; similar findings regarding the effects of biological sex were observed with secukinumab [6], upadactinib [8], and apremilast [9]. Additionally, disease characteristics, such as disease duration, CRP level, and functional status are predictors of response for PsA patients treated with TNFi [4, 5].

Guselkumab, a fully human monoclonal antibody targeting the IL-23p19 subunit, is approved for the treatment of adults with moderate-to-severe psoriasis and/or active PsA. In the Phase 3, randomized, placebo-controlled DISCOVER-1 (1 year) and DISCOVER-2 (2 years) studies, guselkumab demonstrated significantly greater efficacy in improving PsA signs and symptoms across multiple domains versus placebo [10, 11]. Additionally, the proportions of guselkumab-treated patients achieving meaningful responses across PsA domains were sustained or increased through up to 2 years [12, 13]. Of note, post hoc analyses of DISCOVER-2 found that among patients who achieved ≥ 70% improvement in the ACR response criteria (ACR70) or met the composite minimal disease activity (MDA) criteria at 1 year, over 70% and 80%, respectively, maintained this response at 2 years [13]. Post hoc analyses of the pooled DISCOVER-1 and -2 studies further demonstrated that the efficacy of guselkumab through 1 year was consistent across assessed PsA patient subgroups defined by baseline clinical characteristics [14].

Aligning with current treatment guidelines that emphasize tailoring treatment to achieve low levels of disease activity across domains affected in individual patients [2], this post hoc analysis of DISCOVER-2 evaluated patterns of stringent disease control achieved through 2 years, across key GRAPPA-identified domains and patient-reported outcomes (PROs), in subgroups of PsA patients defined by their baseline clinical characteristics.

## Materials and methods

### Study design and patients

Details of study design and patient eligibility criteria were previously described [11]. Briefly, DISCOVER-2 enrolled biologic-naïve adults with active PsA (swollen joint count [SJC] ≥ 5, tender joint count [TJC] ≥ 5, CRP ≥ 0.6 mg/dL) despite standard treatments (csDMARDs, apremilast, or nonsteroidal anti-inflammatory drugs). Patients were randomized (1:1:1) to receive guselkumab 100 mg every 4 weeks (Q4W; *n* = 245); guselkumab 100 mg at Week 0, Week 4, then Q8W (*n* = 248); or placebo (*n* = 246) with crossover to guselkumab 100 mg Q4W at Week 24, with the final study drug administration at Week 100 [11]. The DISCOVER-2 study was conducted in compliance with the Declaration of Helsinki and Good Clinical Practice guidelines; all patients gave written informed consent before any study-related procedures were performed.

### Assessments

Details of the efficacy assessments in the DISCOVER-2 study were previously reported [11]. Physicians assessed TJC (0–68), SJC (0–66), physician global assessment (PhGA; visual analog scale [VAS]; 0–100), enthesitis (Leeds Enthesitis Index [LEI]; 0–6) [15], and dactylitis (Dactylitis Severity Score [DSS]; scale of 0 = no dactylitis to 3 = severe for each digit; total 0–60) [16]. Skin disease was evaluated using the Psoriasis Area and Severity Index (PASI; 0–72) [17] and the Investigator’s Global Assessment (IGA; 0–4) [18]. Patients reported their pain using a VAS (0–10), patient global assessment (PtGA) of arthritis (VAS; 0–100) and arthritis and psoriasis (VAS; 0–100), physical function using the Health Assessment Questionnaire-Disability Index (HAQ-DI; 0–3) [19], and fatigue level using the Functional Assessment of Chronic Illness Therapy (FACIT)-Fatigue (0–52) [20]. The 36-item Short-Form Health Survey (SF-36) was used to evaluate health-related quality of life (HRQoL) [21]. Owing to few reported adverse events (AEs) related to IBD and uveitis [13], axial disease assessments collected in a limited number of patients [22], and no nail psoriasis evaluation performed in DISCOVER-2, these related conditions/domains were not included in the present analysis.

### Outcomes

PsA Disease Activity Score (PASDAS) scores were calculated from the PhGA, PtGA (arthritis and psoriasis), SF-36 physical component summary (PCS) score, SJC, TJC, LEI, dactylitis count (0–20), and CRP [23]. Total PASDAS scores range from 0–10, with low disease activity (LDA) defined as a score ≤ 3.2. MDA is defined as meeting ≥ 5 of the following criteria: TJC ≤ 1, SJC ≤ 1, tender entheseal points ≤ 1, PASI ≤ 1, patient-reported pain ≤ 15, PtGA (arthritis and psoriasis) ≤ 20, and HAQ-DI ≤ 0.5 [1]. The proportion of patients achieving the following endpoints at Weeks 24, 52, and 100 were determined: ≥ 50%/ ≥ 70% improvement in ACR response criteria (ACR50/70) [24]; 100% improvement from baseline in PASI (PASI 100) and IGA 0 (complete skin clearance) among patients with ≥ 3% body surface area (BSA) affected by psoriasis and IGA ≥ 2 at baseline; resolution of dactylitis among patients affected at baseline per DSS; resolution of enthesitis among patients with baseline enthesitis per LEI; HAQ-DI response (≥ 0.35-point improvement from baseline) [25] among patients with a baseline score ≥ 0.35; FACIT-Fatigue response (≥ 4-point improvement from baseline) [26] among patients with FACIT-Fatigue ≤ 48 at baseline; PASDAS LDA; and MDA.

### Statistical methods

In these post hoc analyses, response rates for achieving the aforementioned clinical efficacy endpoints at Week 24 previously reported by treatment group [11, 27] were compared using odds ratios (ORs) and 95% confidence intervals (CIs) to assess the likelihood of achieving response in each guselkumab group versus placebo. Comparisons were made for all patients and within each subgroup defined by the baseline characteristic of interest: i.e., sex; body mass index (BMI; < 25, ≥ 25 to < 30, ≥ 30 kg/m^2^), SJC (< 10, 10 to 15, > 15); TJC (< 10, 10 to 15, > 15); PsA duration (time since diagnosis < 1, ≥ 1 to < 3, ≥ 3 years); CRP (< 1, ≥ 1 to < 2, ≥ 2 mg/dL); PASI (< 12, ≥ 12 to < 20, ≥ 20); BSA (< 3, ≥ 3 to < 10, 10 to < 20, ≥ 20%); concomitant use of any csDMARD; and concomitant use of methotrexate (MTX). ORs and 95% CIs were determined for each baseline patient subgroup at Week 24 using a logistic regression model with treatment group as the explanatory factor. In addition, response rates for patients randomized to the Q4W and Q8W groups were determined at Weeks 52 and 100 for the same baseline characteristic subgroups previously described. At all timepoints, patients with missing data were considered nonresponders (nonresponder imputation [NRI]). All analyses were performed using the statistical software package SAS 9.4 (Statistical Analysis System, SAS-Institute, Cary, NC, USA).

## Results

### Baseline Characteristics

Among the 739 patients randomized and treated in DISCOVER-2, 493 were randomized to guselkumab (245 Q4W; 248 Q8W) and 246 to placebo [11]. Of the 493 guselkumab-randomized patients included in the analysis, 442 (90%) completed study treatment through Week 100 [13]. Baseline clinical characteristics (Table 1) were generally consistent across the randomized treatment groups, except more males comprised the guselkumab Q4W (58%) and Q8W (52%) groups than the placebo (48%) group. Active PsA in this study population was documented by mean numbers of involved joints, levels of pain, and degree of physical function impairment. DISCOVER-2 patients randomized to guselkumab Q4W, guselkumab Q8W, and placebo, respectively, had a mean range for SJC of 11.7–12.9; TJC of 19.8–22.4; PASI of 9.3–10.8; BSA of 17.0%–18.2%; PASDAS of 6.6 in each group; HAQ-DI of 1.2–1.3; and FACIT-Fatigue score of 29.1–30.8. Among patients with enthesitis (68%) or dactylitis (45%) at baseline, mean LEI scores were 2.6–3.0 and mean DSS scores were 8.0–8.6, respectively, across treatment groups. Approximately 70% of patients received concomitant csDMARDs at baseline, with 57%–63% receiving concomitant MTX.

**Table 1:** Baseline demographic and disease characteristics in DISCOVER-2

	Guselkumab 100 mg Q4W	Guselkumab 100 mg Q8W	Placebo
Randomized and treated patients, N	245	248	246
Demographics			
Age, years	45.9 (11.5)	44.9 (11.9)	46.3 (11.7)
Female, n (%)	103 (42)	119 (48)	129 (52)
Male, n (%)	142 (58)	129 (52)	117 (48)
BMI, kg/m^2^	29.1 (5.9)	28.7 (6.3)	29.0 (6.4)
PsA characteristics			
PsA disease duration, years	5.5 (5.9)	5.1 (5.5)	5.8 (5.6)
CRP, mg/dL	1.2 (0.6–2.3)	1.3 (0.7–2.5)	1.2 (0.5–2.6)
PASDAS score (0–10)	6.6 (1.1)	6.6 (1.1)	6.6 (0.9)
SJC (0–66)	12.9 (7.8)	11.7 (6.8)	12.3 (6.9)
TJC (0–68)	22.4 (13.5)	19.8 (11.9)	21.6 (13.1)
Patients with enthesitis, n (%)	170 (69)	158 (64)	178 (72)
Leeds enthesitis index score (1–6)^a^	3.0 (1.7)	2.6 (1.5)	2.8 (1.6)
Patients with dactylitis, n (%)	121 (49)	111 (45)	99 (40)
Dactylitis score (1–60)^b^	8.6 (9.6)	8.0 (9.6)	8.4 (9.3)
Patients with axial involvement, n (%)^c^	82 (33.5)	68 (27.4)	96 (39.0)
History of IBD	1 (0.4)	0	0
History of uveitis	3 (1.2)	1 (0.4)	4 (1.6)
PASI score (0–72)	10.8 (11.7)	9.7 (11.7)	9.3 (9.8)
BSA (0–100%)	18.2 (20.0)	17.0 (21.0)	17.1 (20.0)
IGA score ≥ 3 (0–4), n (%)	117 (48)	108 (44)	115 (47)
HAQ-DI score (0–3)	1.2 (0.6)	1.3 (0.6)	1.3 (0.6)
FACIT-Fatigue score (0–52)	30.8 (9.6)	29.3 (9.9)	29.1 (9.5)
Concomitant medications, n (%)			
csDMARDs	170 (69)	170 (69)	172 (70)
MTX	146 (60)	141 (57)	156 (63)
Oral corticosteroids	46 (19)	50 (20)	49 (20)

Data are presented as mean (SD) or median (IQR) unless otherwise noted. ^a^Among patients with enthesitis and available Leeds Enthesitis Index score at baseline (Q4W group *n* = 166, Q8W *n* = 157, and placebo group *n* = 175). ^b^Among patients with available dactylitis score at baseline (Q4W group *n* = 121, Q8W *n* = 111, and placebo group *n* = 99). ^c^Investigator-verified, imaging-confirmed sacroiliitis. BMI: body mass index; BSA: body surface area; CRP: C-reactive protein; csDMARD: conventional synthetic disease-modifying antirheumatic drug; FACIT: Functional Assessment of Chronic Illness Therapy; HAQ-DI: Heath Assessment Questionnaire-Disability Index; IBD: inflammatory bowel disease; IGA: Investigator’s Global Assessment of psoriasis; IQR: interquartile range; MTX: methotrexate; PASDAS: Psoriatic Arthritis Disease Activity Score; PASI: Psoriasis Area and Severity Index; PsA: psoriatic arthritis; Q4W: every 4 weeks; Q8W: every 8 weeks; SD: standard deviation; SJC: swollen joint count; TJC: tender joint count

### Substantial Improvements in Joint Disease

At Week 24, 33% of patients in the Q4W group and 31% in the Q8W group achieved ACR50 response compared with 14% in the placebo group; 13%, 19%, and 4%, respectively, achieved ACR70 response. The corresponding ORs for achieving these responses with guselkumab Q4W and Q8W, respectively, were 3.0 and 2.8 for ACR50, and 3.5 and 5.4 for ACR70 (Supplementary Fig. S1). Response patterns were consistent, with higher likelihood of achievement with guselkumab versus placebo, across patient subgroups with adequate sample size.

**Fig. 1:**
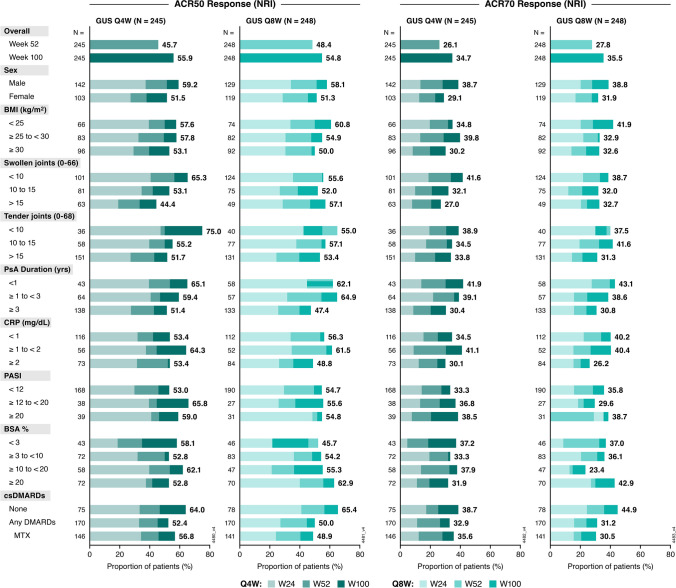
Proportions of guselkumab-randomized patients achieving ACR50 and ACR70 response through Week 100 by baseline characteristics. Proportions displayed for subgroups indicate the response rates (utilizing NRI) at Week 100. ACR50: ≥ 50% improvement in American College of Rheumatology response criteria; ACR70: ≥ 70% improvement in American College of Rheumatology response criteria; BMI: body mass index; BSA: body surface area; CRP: C-reactive protein; csDMARD: conventional synthetic disease-modifying antirheumatic drug; GUS: guselkumab; MTX: methotrexate; NRI: nonresponder imputation; PASI: Psoriasis Area and Severity Index; PsA: psoriatic arthritis; Q4W: every 4 weeks; Q8W: every 8 weeks; W: Week

ACR50 and ACR70 response rates increased over time in nearly all subgroups, including females; patients with BMI ≥ 30; and those with more active disease as assessed by SJC, TJC, and % BSA affected by psoriasis. Across most patient subgroups, ACR50 response was generally achieved by a majority of patients (44%–75%) and ACR70 response was achieved by more than one-third (23%–45%) of guselkumab-treated patients through Week 100 (Fig. 1).

### Complete skin clearance

The 543 patients with ≥ 3% BSA with psoriasis and IGA ≥ 2 at baseline were included in analyses of skin response. Greater proportions of guselkumab- than placebo-treated patients achieved PASI 100 (45% versus 3%) or IGA 0 (50%–51% versus 8%) at Week 24, with corresponding OR ranges of 28.6 to 29.7 for PASI 100 and 12.1 to 12.3 for IGA 0 (Supplementary Fig. S2). The treatment effect of guselkumab was consistent across all patient subgroups, with corresponding ORs for achieving complete skin clearance with either guselkumab dosing regimen ranging from 11.7 to 80.6 for PASI 100 and 5.3 to 56.2 for IGA 0.

**Fig. 2:**
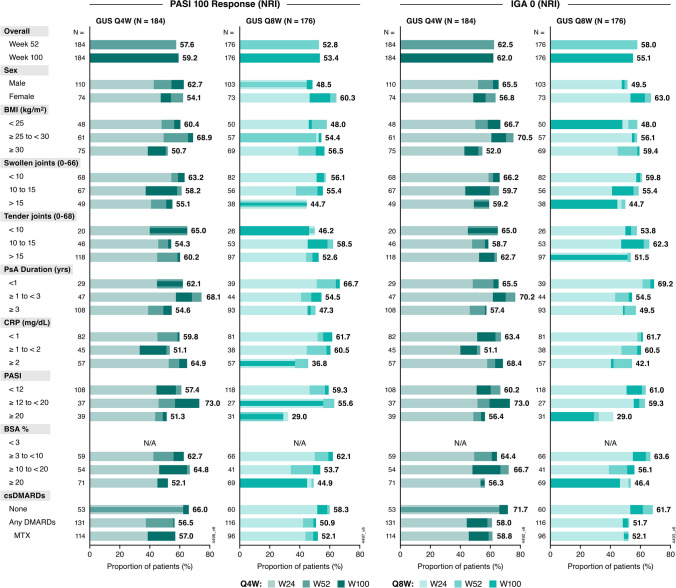
Proportions of guselkumab-randomized patients achieving PASI 100 and IGA 0 through Week 100 by baseline characteristics. Achievement of PASI 100 and IGA 0 was assessed among patients with BSA ≥ 3% and IGA ≥ 2 at baseline. Proportions displayed for subgroups indicate the response rates (utilizing NRI) at Week 100. BMI: body mass index; BSA: body surface area; CRP: C-reactive protein; csDMARD: conventional synthetic disease-modifying antirheumatic drug; GUS: guselkumab; IGA: Investigator’s Global Assessment; MTX: methotrexate; NRI: nonresponder imputation; PASI: Psoriasis Area and Severity Index; PASI 100: 100% improvement in PASI; PsA: psoriatic arthritis; Q4W: every 4 weeks; Q8W: every 8 weeks; W: Week

PASI 100 and IGA 0 response rates were sustained or increased over time. A majority of guselkumab-treated patients (50%–70%) in most subgroups achieved these stringent levels of response, indicating clear skin, through Week 100 (Fig. 2).

### Dactylitis and enthesitis resolution

Among patients with dactylitis at baseline, 64% in the Q4W group and 57% in the Q8W group achieved resolution at Week 24, compared with 38% of patients in the placebo group. Importantly, this effect was generally consistent across all baseline subgroups of adequate size. Similar trends were observed when evaluating resolution of enthesitis among patients affected at baseline: 44% of patients in the Q4W group and 54% in the Q8W group achieved enthesitis resolution at Week 24, compared with 30% of those in the placebo group (Supplementary Fig. S3).

**Fig. 3:**
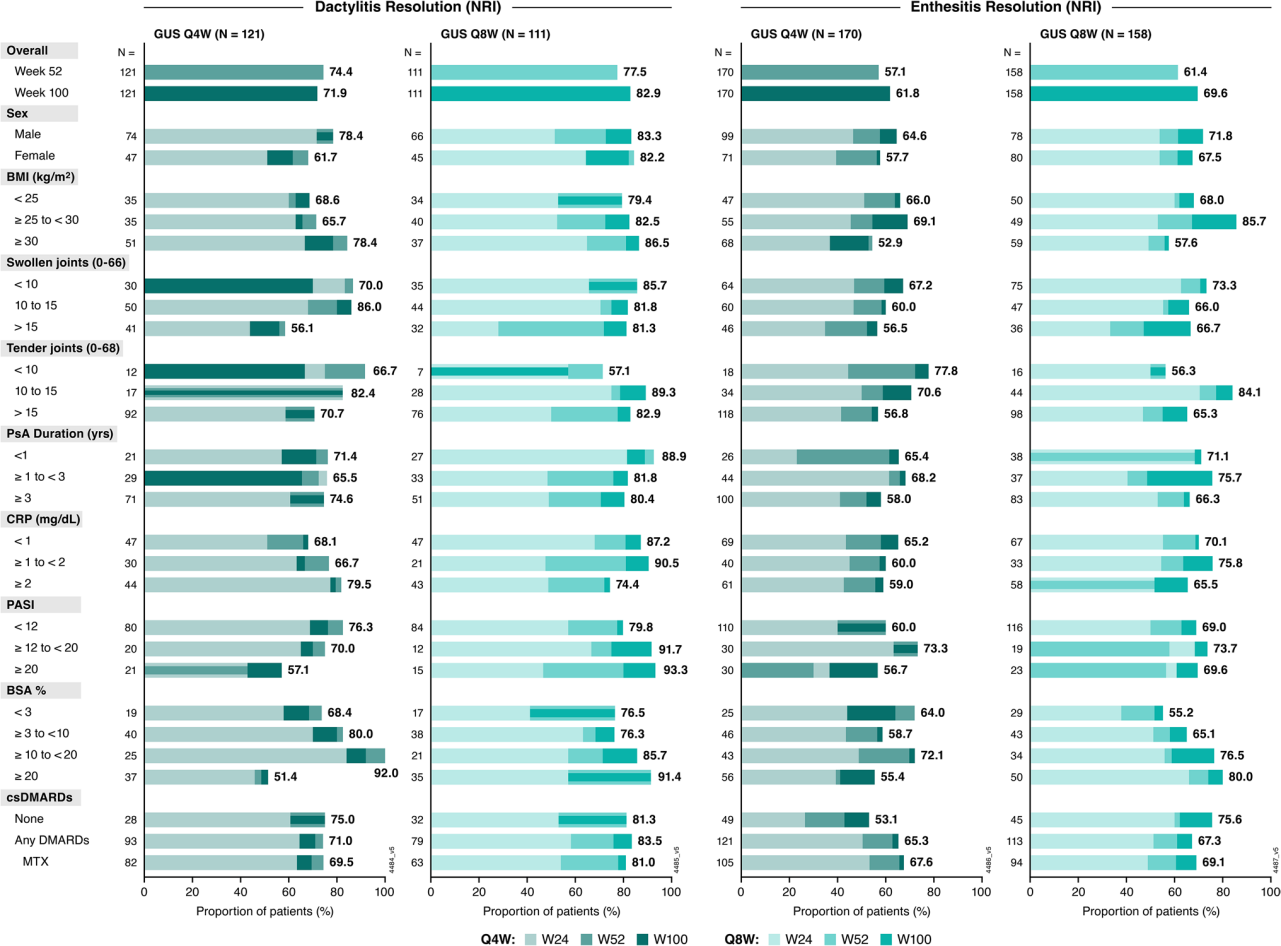
Proportion of guselkumab-randomized patients achieving resolution of dactylitis and enthesitis through Week 100 by baseline characteristics. Resolution of dactylitis and enthesitis was assessed among patients with dactylitis and enthesitis at baseline, respectively. Proportions displayed for subgroups indicate the response rates (utilizing NRI) at Week 100. BMI: body mass index; BSA: body surface area; CRP: C-reactive protein; csDMARD: conventional synthetic disease-modifying antirheumatic drug; GUS: guselkumab; MTX: methotrexate; NRI: nonresponder imputation; PASI: Psoriasis Area and Severity Index; PsA: psoriatic arthritis; Q4W: every 4 weeks; Q8W: every 8 weeks; W: Week

Rates of dactylitis resolution among guselkumab-randomized patients generally increased through Week 52 and were maintained or increased thereafter regardless of baseline subgroup. Similarly, the proportions of patients with enthesitis resolution at Week 24 increased through Week 100 at the population level, except in the subgroup of patients with BSA < 3% in the Q4W regimen for which response peaked at Week 52 (Fig. 3).

Complete resolution of dactylitis and enthesitis at Week 100 was achieved by the majority of guselkumab-randomized patients in baseline subgroups of adequate size. In several baseline subgroup categories, most notably those with more extensive skin psoriasis (BSA ≥ 3% to < 20%), ≥ 80% of guselkumab-randomized patients achieved dactylitis resolution. Similarly, approximately two-thirds of patients achieved enthesitis resolution in most subgroups, surpassing 70% in several categories (Fig. 3).

### Clinically meaningful improvements in patient-reported outcomes

**Fig. 4:**
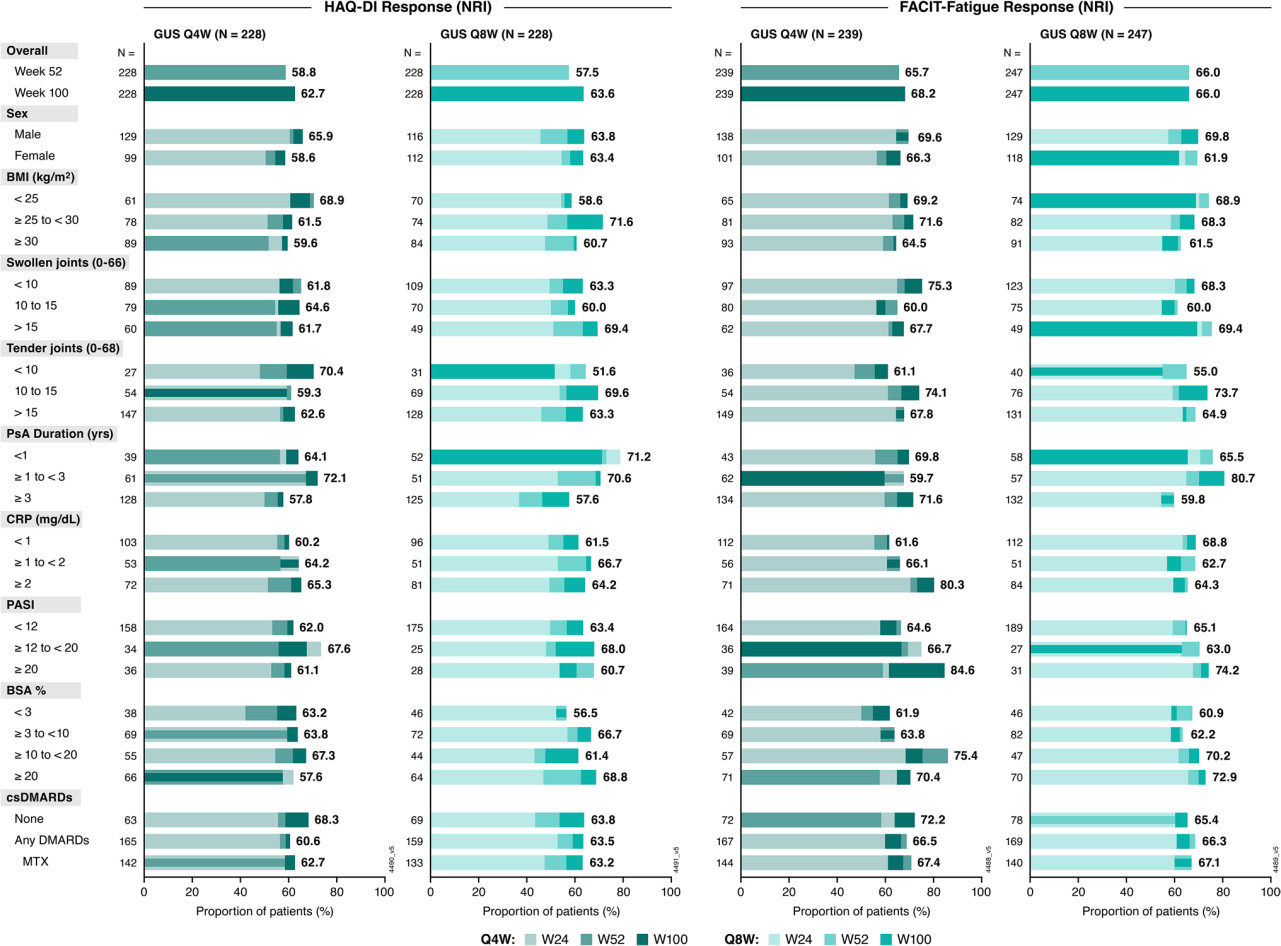
Proportion of guselkumab-randomized patients achieving HAQ-DI and FACIT-Fatigue response through Week 100 by baseline characteristics. Achievement of HAQ-DI response (improvement ≥ 0.35) FACIT-Fatigue response (improvement ≥ 4) was assessed among patients with baseline HAQ-DI ≥ 0.35 and baseline FACIT-Fatigue ≤ 48, respectively. Proportions displayed for subgroups indicate the response rates (utilizing NRI) at Week 100. BMI: body mass index; BSA: body surface area; CRP: C-reactive protein; csDMARD: conventional synthetic disease-modifying antirheumatic drug; FACIT: Functional Assessment of Chronic Illness Therapy; GUS: guselkumab; HAQ-DI: Health Assessment Questionnaire-Disability Index; MTX: methotrexate; NRI: nonresponder imputation; PASI: Psoriasis Area and Severity Index; PsA: psoriatic arthritis; Q4W: every 4 weeks; Q8W: every 8 weeks; W: Week

At Week 24, patients who received either guselkumab regimen were significantly more likely to achieve a HAQ-DI response (≥ 0.35-point improvement from baseline among patients with HAQ-DI ≥ 0.35 at baseline) than placebo-treated patients, both overall and across all baseline subgroups, with ORs ranging from 1.2 to 18.8 (Supplementary Fig. 4). Response rates were generally enhanced through Week 100. Within each baseline subgroup, a majority of guselkumab-randomized patients reported clinically meaningful improvements in activities of daily living at Week 100, with response rates generally ranging between 60% and 70% (Fig. 4).

Likewise, higher proportions of guselkumab- than placebo-treated patients achieved FACIT-Fatigue response (≥ 4-point improvement from baseline among patients with FACIT-Fatigue ≤ 48 at baseline) at Week 24, regardless of regimen, both in the total population and across all baseline subgroups, except those in the lowest categories of TJC (< 10) and BSA (< 3%) (Supplementary Fig. S4). Among guselkumab-randomized patients, FACIT-Fatigue response rates at Week 52 were maintained or increased through Week 100 in most baseline subgroups of adequate size, with substantial proportions (60%–75%) achieving FACIT-Fatigue response at Week 100. In several baseline subgroup categories, including PsA duration ≥ 1 to < 3 years (Q8W), PASI ≥ 20 (Q4W), and CRP ≥ 2 mg/dL (Q4W), ≥ 80% of patients receiving guselkumab reported meaningful improvements in fatigue (Fig. 4).

### Overall disease activity

**Fig. 5:**
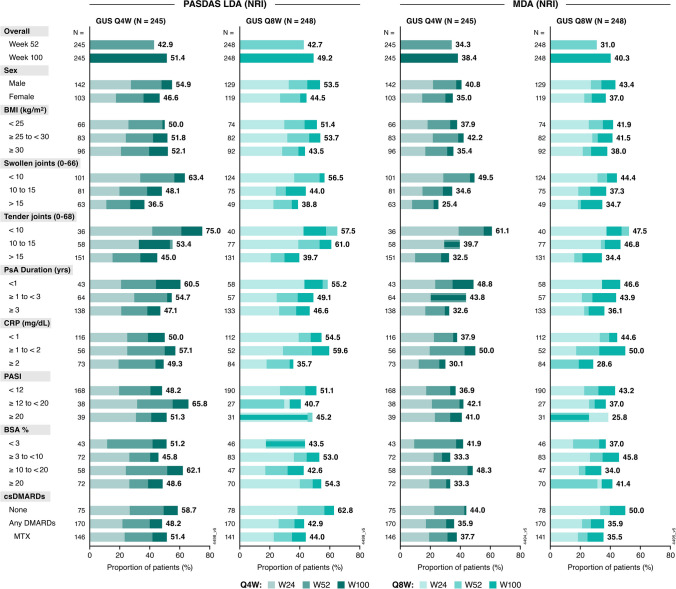
Proportion of guselkumab-randomized patients achieving PASDAS LDA and MDA through Week 100 by baseline characteristics. Proportions displayed for subgroups indicate the response rates (utilizing NRI) at Week 100. BMI: body mass index; BSA: body surface area; CRP: C-reactive protein; csDMARD: conventional synthetic disease-modifying antirheumatic drug; GUS: guselkumab; LDA: low disease activity; MDA: minimal disease activity; MTX: methotrexate; NRI: nonresponder imputation; PASDAS: Psoriatic Arthritis Disease Activity Score; PASI: Psoriasis Area and Severity Index; PsA: psoriatic arthritis; Q4W: every 4 weeks; Q8W: every 8 weeks; W: Week

Greater proportions of guselkumab- than placebo-treated patients achieved PASDAS LDA overall and across all baseline subgroups (Supplementary Fig. S5). Rates of PASDAS LDA achievement generally increased through Week 52 and again through Week 100 regardless of baseline subgroup, with response rates generally ranging from 40% to 65% at Week 100 (Fig. 5).

As with PASDAS LDA and consistent with individual PsA domains assessed, higher proportions of guselkumab- than placebo-treated patients achieved MDA at Week 24, and the benefit of guselkumab versus placebo was observed in all baseline subgroups, except in patients with minimal skin disease at baseline (Supplementary Fig. S5). Substantial increases in MDA response rates were observed among guselkumab-randomized patients through Weeks 52 and 100 at the population level, a pattern generalizable to nearly all subgroups. Considerable proportions (generally ranging from 35%–50%) of guselkumab-randomized patients in each baseline subgroup achieved MDA at Week 100 (Fig. 5).

## Discussion

The present analyses provide evidence to inform the clinical use of guselkumab in patients across a range of baseline characteristics, and in multiple PsA disease domains. Current treatment guidelines support achievement of low levels of disease activity in PsA domains affected in individual patients [2]. A pooled analysis of the DISCOVER-1 and -2 trials showed that, on average, guselkumab-treated patients (Q4W and Q8W) achieved greater improvement versus placebo in PsA signs and symptoms at Week 24, irrespective of baseline demographic and clinical characteristics, or concomitant csDMARDs (including MTX) [14]. In the current analyses utilizing data from biologic-naïve patients with active PsA in DISCOVER-2, the treatment effect of guselkumab at Week 24 was consistent with the pooled analyses. Further, guselkumab treatment was associated with durable achievement of stringent endpoints through up to 2 years, across key GRAPPA-identified disease domains and PROs, regardless of baseline characteristics.

Across baseline subgroups, over one-half of guselkumab-randomized patients achieved an ACR50 response and more than one-third achieved an ACR70 response at Week 100, indicating notable improvements in peripheral joint disease. Furthermore, 50%–70% of guselkumab-randomized patients achieved complete skin clearance, which is associated with meaningful differences in HRQoL and pain improvement among patients with psoriasis, even when considering achievement of a less stringent PASI 90 response [28–30]. Consistently, in the VOYAGE trials, patients with moderate-to-severe plaque psoriasis achieved high levels of response, maintained through 5 years of guselkumab therapy [31]. Among patients in the guselkumab groups with dactylitis or enthesitis at baseline, response rates for dactylitis and enthesitis resolution at Week 100 ranged from 65%–85%, regardless of baseline characteristics. Previous pooled analyses of DISCOVER-1 and -2 found that resolution of these hallmark manifestations of PsA was associated with achieving minimal TJC/SJC and MDA [32, 33]. Both guselkumab regimens were also shown to provide early and durable improvements across key GRAPPA-recognized PsA domains through 2 years of treatment, with substantial proportions of patients achieving low/minimal levels of overall disease activity including peripheral arthritis, enthesitis, dactylitis, axial symptoms, and skin psoriasis [34].

Consistent response patterns were observed for PROs. Approximately 60%–80% of guselkumab-randomized patients across all subgroups achieved clinically meaningful improvements in physical function and fatigue through Week 100, both of which have been prioritized by patients for evaluating treatment options in clinical trials [35].

Notable proportions of guselkumab-randomized patients achieved low or minimal levels of disease activity through 2 years, regardless of baseline characteristics, as assessed by composite indices accounting for peripheral joint disease and extra-articular domains. Specifically, in subgroups of adequate size, > 40% of patients in the Q4W and Q8W groups achieved PASDAS LDA and over one-third achieved MDA by Week 100. It is noteworthy that response rates for achieving PASDAS LDA and MDA increased over time across subgroups, including those with characteristics that have been associated with reduced efficacy with other PsA treatments (i.e., female sex, obesity, and CRP level) [4, 5, 7], as well as patients with more active skin and joint disease at baseline.

The findings of these post hoc analyses are particularly impactful for the therapeutic management of PsA given its heterogenous nature [2]. The ability of guselkumab to provide durable disease control across key PsA domains and PROs, irrespective of baseline patient and clinical characteristics or concomitant csDMARD use, indicates it is an effective treatment option for diverse PsA patient types. In particular, the pattern of response rates over time, with increasing proportions of variable patient phenotypes achieving meaningful levels of disease control, may reduce the need for continuous treatment optimization and encourage treatment persistence. In DISCOVER-2, 90% of guselkumab-randomized patients completed study treatment through 2 years. In real-world settings, patients with PsA receiving guselkumab were 3 times more likely than those receiving an initial subcutaneous TNFi to remain on therapy through 12 months [36], and guselkumab had the highest drug survival associated with effectiveness compared with several other biologics used by registry participants with psoriasis [37].

The durable efficacy of guselkumab across key PsA domains and PROs in an array of patient phenotypes also reinforces the critical role played by selective IL-23p19 inhibition in the treatment of the underlying PsA pathophysiology. In previous analyses from the DISCOVER studies, guselkumab therapy was associated with significant decreases from baseline in markers of inflammation, including Th17 effector cytokines [38], with sustained pharmacodynamic effects observed through 2 years in DISCOVER-2 [39]. The durable and substantial multi-domain efficacy of guselkumab may also be ascribed to its distinct molecular attributes, i.e., the ability to bind CD64 expressed by IL-23-producing myeloid cells, thereby blocking IL-23 at the cellular source of inflammation [40].

Although safety was not evaluated in the current analyses, guselkumab has been shown to have a favorable profile comparable to placebo, with the types and frequencies of AEs remaining consistent through up to 2 years, regardless of prior TNFi therapy, in a pooled analysis from four Phase 2/3 studies of 1,554 TNFi-naïve and -experienced patients with active PsA [41]. Rates per 100 patient-years of AEs leading to treatment discontinuation, serious AEs and other AEs of interest were low and comparable between the placebo and guselkumab groups through 24 weeks of treatment, and between the Q4W and Q8W dosing regimens during long-term evaluation [41]. Owing to small sample sizes, PsA-related conditions of IBD and uveitis were not assessed in the present analysis. However, among guselkumab-treated patients included in the pooled safety analysis of PsA studies, there were few reports of these AEs, with one suspected case of IBD and one case of uveitis [41]. In addition, consistent safety results have been observed across patients subgroups defined by sex, age, BMI, and prior TNFi therapy in an integrated analysis of 11 Phase 2 and 3 studies of psoriasis and PsA patients receiving guselkumab for up to 5 years [42]. The established long-term safety profile, together with the results of the current analysis, support the long-term favorable benefit-to-risk ratio of guselkumab in treating diverse groups of patients with active PsA.

This was a post hoc analysis of the DISCOVER-2 trial that was not powered to detect treatment effects across patient subgroups, some of which contained relatively small numbers of patients. Results reported here through 2 years are strengthened by the high patient retention rate in DISCOVER-2, with 90% of guselkumab-randomized patients completing treatment through Week 100. As no associations between baseline characteristics and discontinuation were anticipated, this analysis was not performed. Assessments of nail psoriasis were not performed in the DISCOVER-2 study and sample size limitations precluded inclusion of axial disease in the present analysis. However, the efficacy of guselkumab in nail disease derived from psoriasis patients with self-reported PsA in the VOYAGE 1 and 2 trials has recently been reported [43]. Additionally, post hoc analyses of DISCOVER-2 showed that guselkumab improved axial symptoms through 2 years among patients with imaging-confirmed sacroiliitis [22]. In addition, exploratory subgroup analyses demonstrated a consistent guselkumab treatment effect in both male and female patients [22]. Due to more restrictive enrollment criteria in clinical trials, these results may have limitations when considering a broader population of PsA patients seen in clinical practice. Evidence from real-world studies may prove useful to pragmatically assess PsA patients not reflected in DISCOVER-2. To that end, a recent analysis utilized data from the CorEvitas PsA/Spondyloarthritis Registry to evaluate the effectiveness of guselkumab among PsA patients with persistent treatment (Q8W) for 6 months [44]. Despite these patients having largely longstanding and treatment-resistant disease (73% with ≥ 2 prior biologic/targeted synthetic DMARDs), statistically significant improvements from baseline in the clinical Disease Activity Index for PsA (cDAPSA) were observed at 6 months, with 25% of those with moderate-to-severe disease at baseline achieving LDA or remission. Results from the ongoing, international, prospective, observational PsABIOnd study will also provide valuable insight into the long-term effectiveness of guselkumab in routine clinical practice [45].

In summary, substantial proportions of adults with active PsA, who received up to 2 years of guselkumab in DISCOVER-2, showed durable achievement of stringent endpoints across key PsA disease domains and PROs. These outcomes included 50%-70% improvement in signs and symptoms of peripheral joint disease, skin clearance, dactylitis and enthesitis resolution, clinically meaningful improvements in physical function and fatigue, and low levels of overall disease activity, regardless of baseline characteristics or guselkumab dosing regimen. Sustained stringent disease control across key PsA domains was achieved at the population level irrespective of sex, BMI, disease activity, or use of concomitant csDMARDs, including MTX. Thus, results of these analyses lend further support to guselkumab as an effective means of tailoring treatment to achieve low levels of disease activity across key domain involvement in individual patients.

### Supplementary Information

Below is the link to the electronic supplementary material.

Supplementary file1 (1.72 MB)

## Data Availability

The data sharing policy of Janssen Pharmaceutical Companies of Johnson & Johnson is available at https://www.janssen.com/clinical-trials/transparency. As noted on this site, requests for access to the study data can be submitted through the Yale Open Data Access (YODA) Project site at http://yoda.yale.edu.
