# Supplementary material for: Durable control of psoriatic arthritis with guselkumab across domains and patient characteristics: post hoc analysis of a phase 3 study
Source: Clin Rheumatol. 2024 Jun 7;43(8):2551–63. doi: 10.1007/s10067-024-06991-8 (PMC11269379; doi:10.1007/s10067-024-06991-8)

# Supplementary Figures

# Supplementary Fig. S1 Proportions of patients achieving ACR50 and ACR 70 responses at Week 24 by baseline demographic and disease characteristics. ACR50: ≥50% improvement in American College of Rheumatology response criteria; ACR70: ≥70% improvement in American College of Rheumatology response criteria; BMI: body mass index; BSA: body surface area; CI: confidence interval; CRP: C-reactive protein; csDMARD: conventional synthetic disease-modifying antirheumatic drug; GUS: guselkumab; MTX: methotrexate; PASI: Psoriasis Area and Severity Index; PBO: placebo; PsA: psoriatic arthritis; Q4W: every 4 weeks; Q8W: every 8 weeks


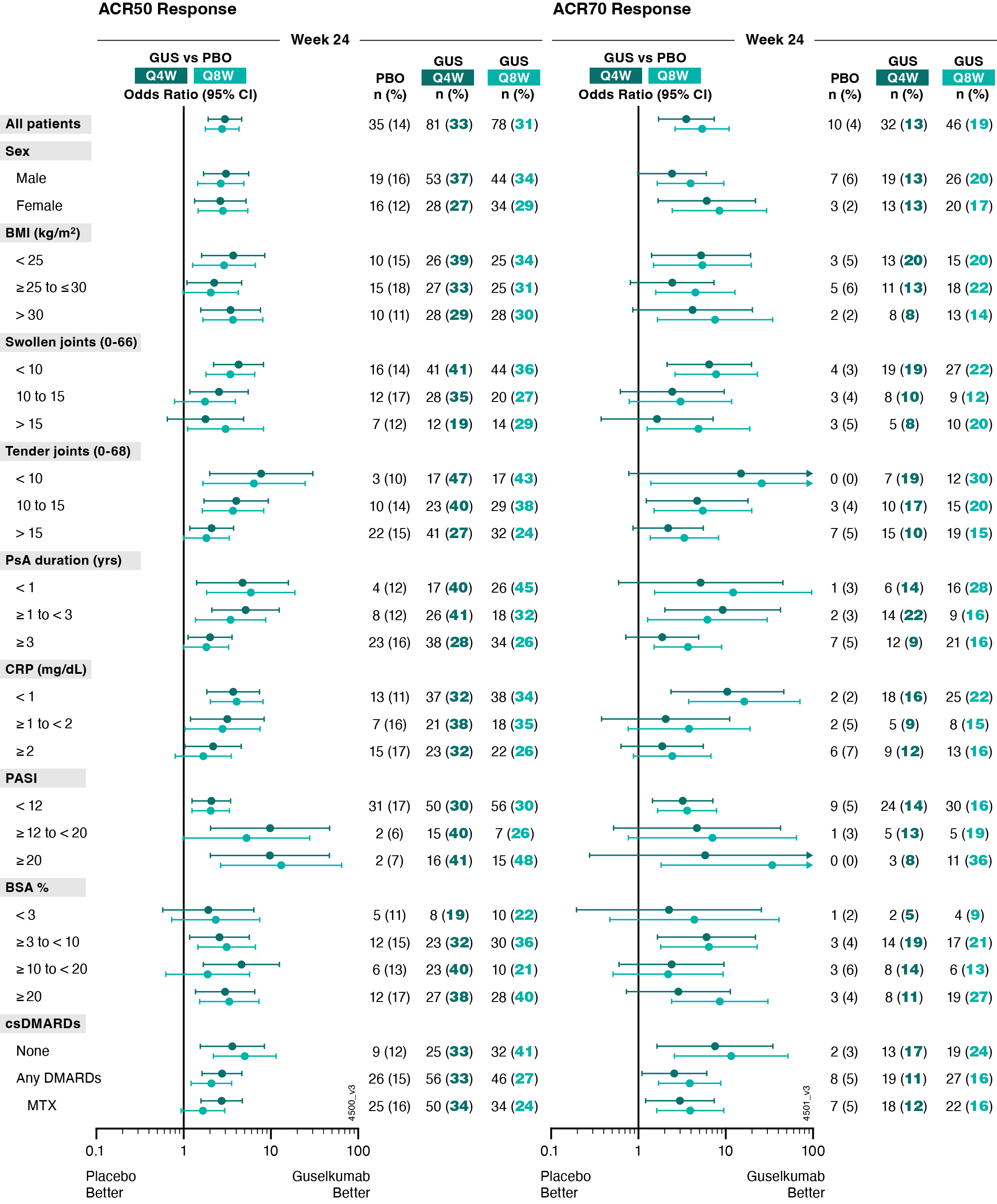


# Supplementary Fig. S2 Proportions of patients achieving PASI 100 and IGA 0 Response (among patients with BSA ≥3% and IGA ≥2 at baseline) at Week 24 by baseline demographic and disease characteristics. BMI: body mass index; BSA: body surface area; CI: confidence interval; CRP: C-reactive protein; csDMARD: conventional synthetic disease-modifying antirheumatic drug; GUS: guselkumab; IGA: Investigator’s Global Assessment; MTX: methotrexate; PASI: in Psoriasis Area and Severity Index; PASI 100: 100% improvement in PASI; PBO: placebo; PsA: psoriatic arthritis; Q4W: every 4 weeks; Q8W: every 8 weeks


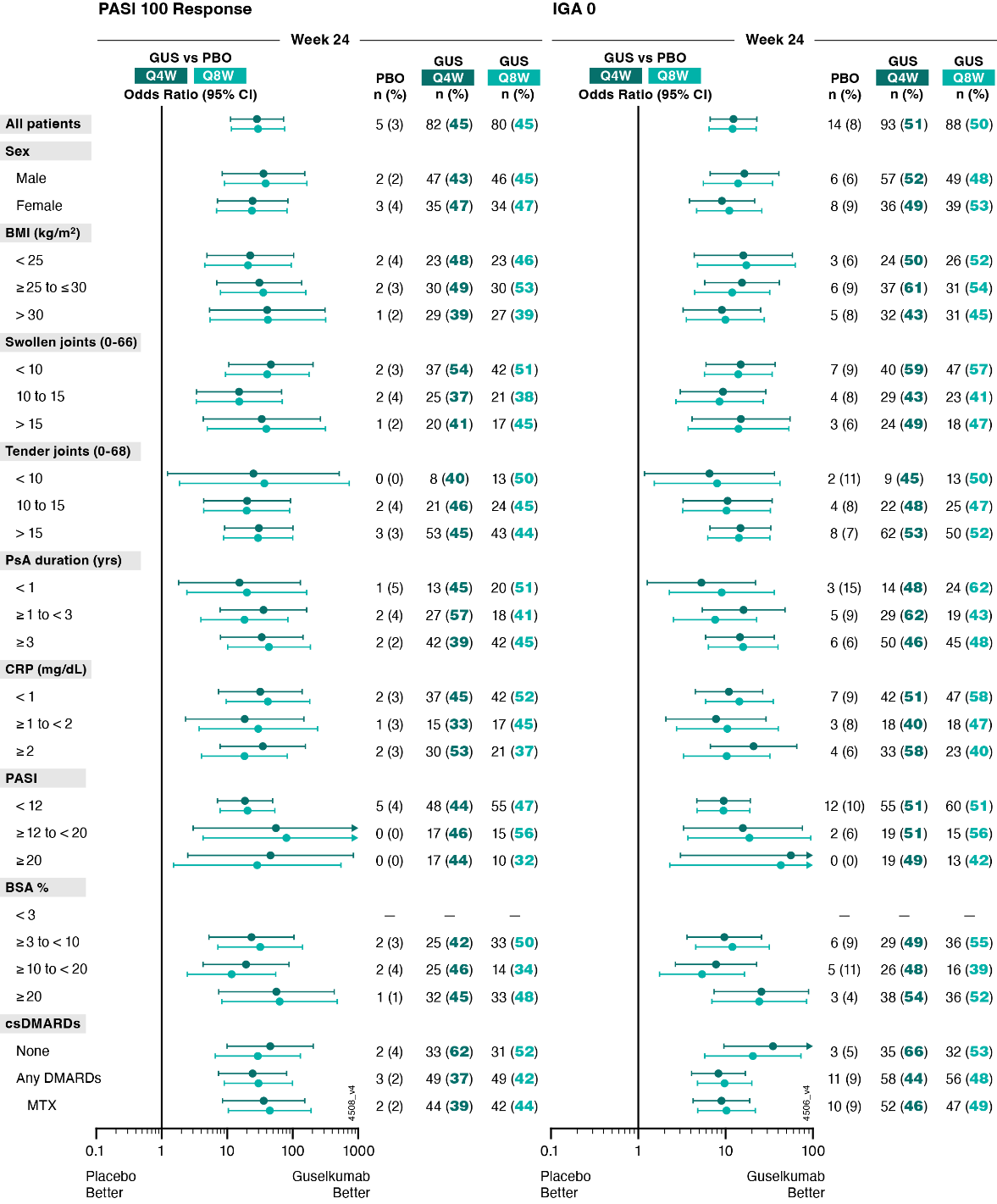


# Supplementary Fig. S3 Proportions of patients achieving resolution of dactylitis and enthesitis (among patients affected at baseline) at Week 24 by baseline demographic and disease characteristics. BMI: body mass index; BSA: body surface area; CI: confidence interval; CRP: C-reactive protein; csDMARD: conventional synthetic disease-modifying antirheumatic drug; GUS: guselkumab; MTX: methotrexate; PASI: Psoriasis Area and Severity Index; PBO: placebo; PsA: psoriatic arthritis; Q4W: every 4 weeks; Q8W: every 8 weeks


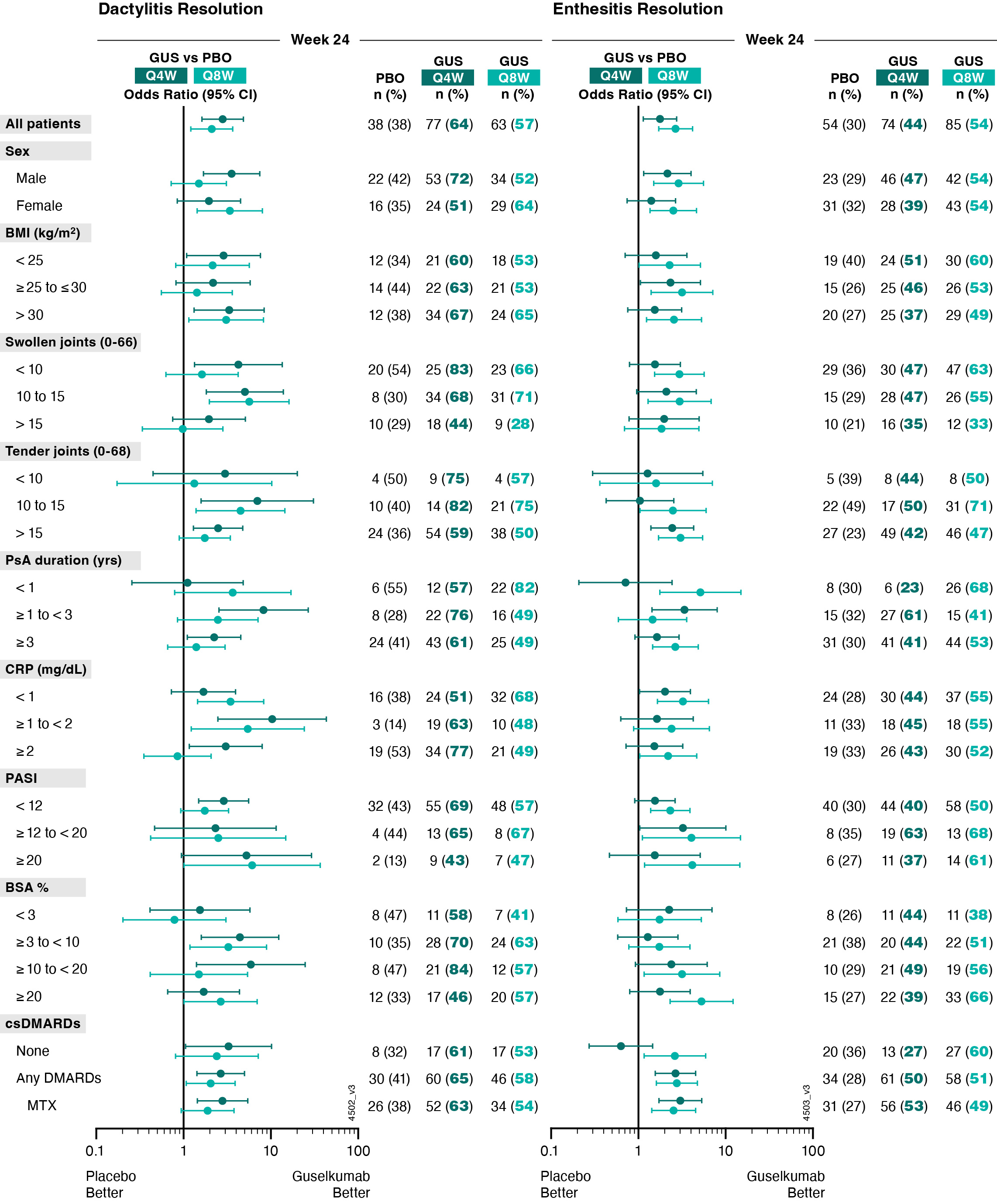


# Supplementary Fig. S4 Proportions of patients achieving HAQ-DI and FACIT-Fatigue responses at Week 24 by treatment group and baseline characteristics. Achievement of HAQ-DI response (improvement ≥0.35) FACIT-Fatigue response (improvement ≥4) was assessed among patients with baseline HAQ-DI ≥0.35 and baseline FACIT-Fatigue ≤48, respectively. BMI: body mass index; BSA: body surface area; CI: confidence interval; CRP: C-reactive protein; csDMARD: conventional synthetic disease-modifying antirheumatic drug; FACIT: Functional Assessment of Chronic Illness Therapy; GUS: guselkumab; HAQ-DI: Health Assessment Questionnaire-Disability Index; MTX: methotrexate; PASI: Psoriasis Area and Severity Index; PBO: placebo; PsA: psoriatic arthritis; Q4W: every 4 weeks; Q8W: every 8 weeks


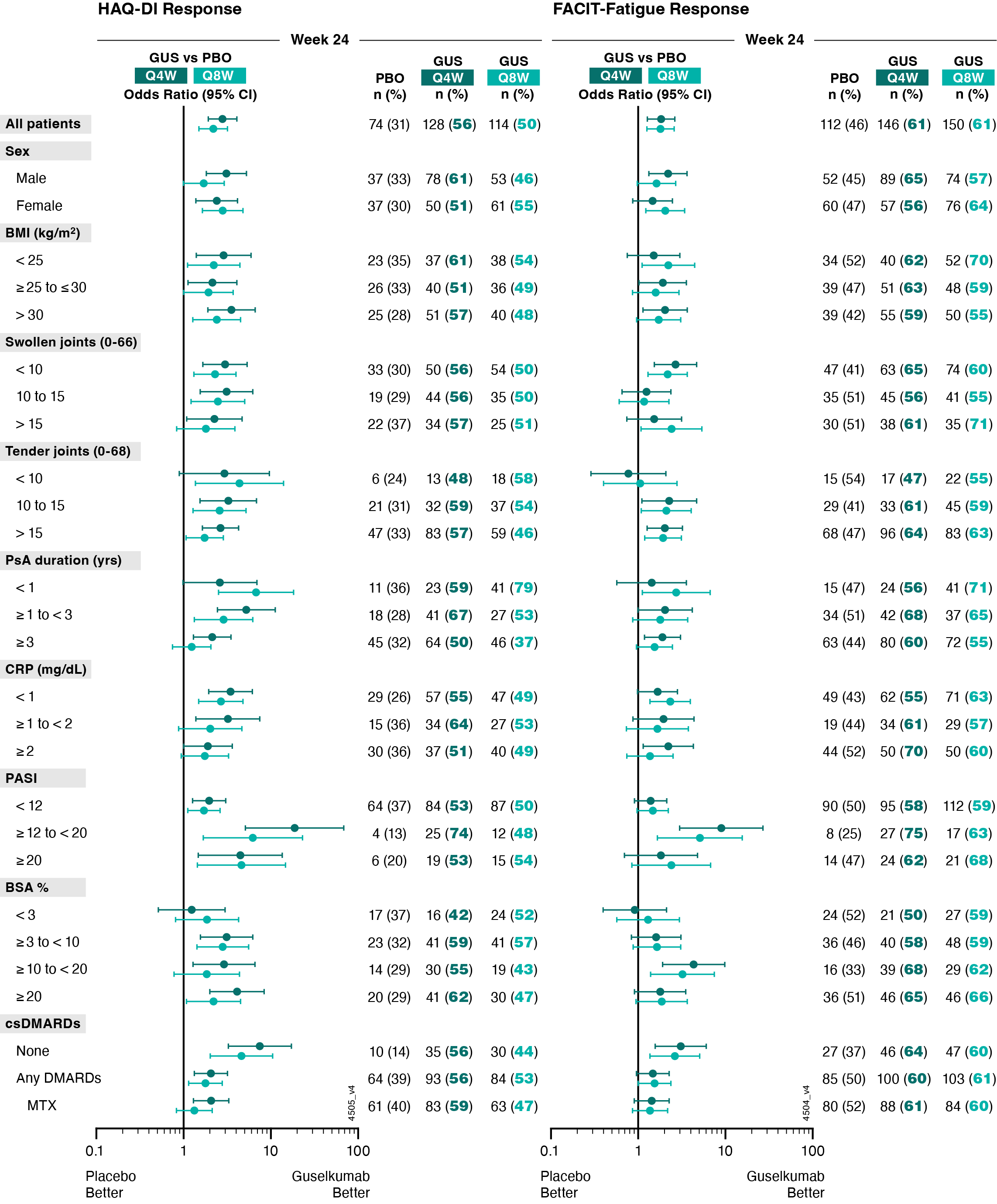


# Supplementary Fig. S5 Proportions of patients achieving PASDAS LDA and MDA at Week 24 by treatment group and baseline characteristics. BMI: body mass index; BSA: body surface area; CI: confidence interval; CRP: C-reactive protein; csDMARD: conventional synthetic disease-modifying antirheumatic drug; GUS: guselkumab; LDA: low disease activity; MDA: minimal disease activity; MTX: methotrexate; PASDAS: Psoriatic Arthritis Disease Activity Score; PASI: Psoriasis Area and Severity Index; PBO: placebo; PsA: psoriatic arthritis; Q4W: every 4 weeks; Q8W: every 8 weeks


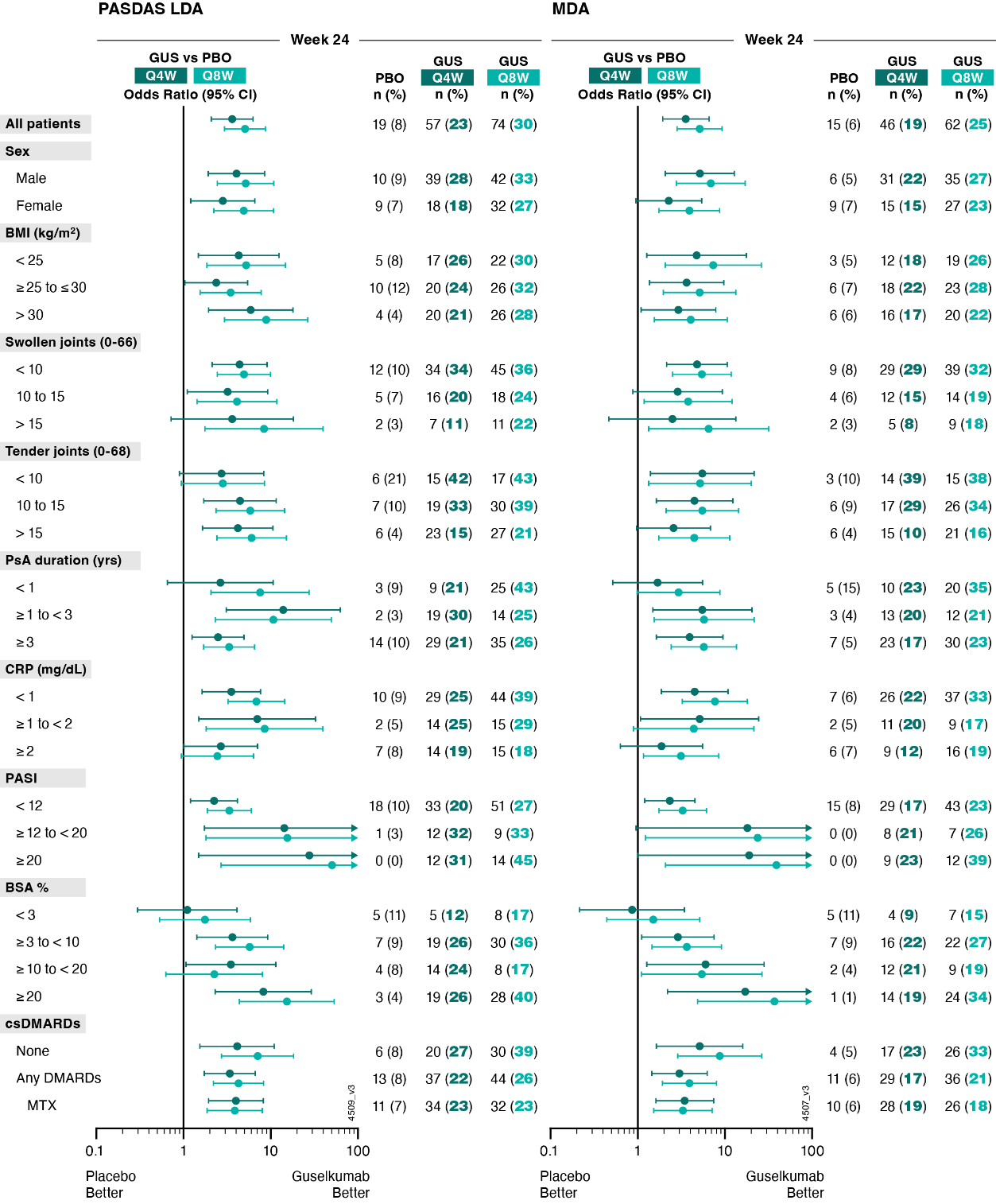

Supplement: Supplementary file 1 — Supplementary file1 (1.72 MB) [file 10067_2024_6991_MOESM1_ESM.docx]
